# Combined *operando* X-ray diffraction–electrochemical impedance spectroscopy detecting solid solution reactions of LiFePO_4_ in batteries

**DOI:** 10.1038/ncomms9169

**Published:** 2015-09-08

**Authors:** Michael Hess, Tsuyoshi Sasaki, Claire Villevieille, Petr Novák

**Affiliations:** 1 Paul Scherrer Institute, Electrochemical Energy Storage Section, 5232 Villigen PSI, Switzerland.; 2 ETH Zurich, Department of Chemistry and Applied Biosciences, Laboratory of Inorganic Chemistry, 8093 Zurich, Switzerland.; 3 Toyota Central R&D Labs., Inc., Battery Laboratory, Nagakute, Aichi 480-1192, Japan.

## Abstract

Lithium-ion batteries are widely used for portable applications today; however, often suffer from limited recharge rates. One reason for such limitation can be a reduced active surface area during phase separation. Here we report a technique combining high-resolution *operando* synchrotron X-ray diffraction coupled with electrochemical impedance spectroscopy to directly track non-equilibrium intermediate phases in lithium-ion battery materials. LiFePO_4_, for example, is known to undergo phase separation when cycled under low-current-density conditions. However, o*perando* X-ray diffraction under ultra-high-rate alternating current and direct current excitation reveal a continuous but current-dependent, solid solution reaction between LiFePO_4_ and FePO_4_ which is consistent with previous experiments and calculations. In addition, the formation of a preferred phase with a composition similar to the eutectoid composition, Li_0.625_FePO_4_, is evident. Even at a low rate of 0.1*C*, ∼20% of the X-ray diffractogram can be attributed to non-equilibrium phases, which changes our understanding of the intercalation dynamics in LiFePO_4_.

## Abstract (web summary)

A clear understanding of the phase behaviour of lithium-ion electrode materials is essential for the development of the field. Here, the authors report a combined X-ray diffraction–electrochemical impedance spectroscopy method to detect solid solution reactions of LiFePO_4_.

## Graphical abstract

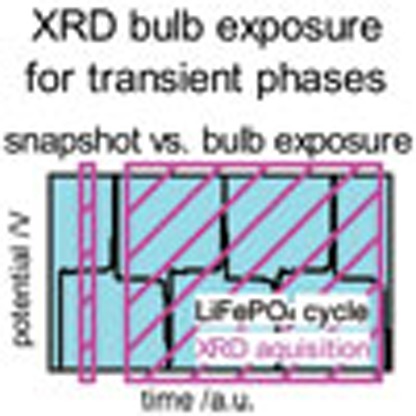

The ability of LiFePO_4_ (LFP) to function as a cathode material in lithium-ion batteries was first reported by Padhi *et al*.^1^ and this material is currently one of the most interesting and most extensively studied lithium-ion battery cathode materials under development^2^,^3^. To make LFP a suitable cathode material for commercial batteries, however, its low electrical conductivity must be increased. Approaches such as nanosizing, carbon-coating^4^, Mg-, Nb-, Ti-, and/or Zr-doping^5^, and Li-pyrophosphate coating^6^ of LFP have resulted in significant enhancements of the charge/discharge rates of up to ∼50*C* for 50-nm particles based on an 80% specific charge^6^ (1*C* equals one full charge in 1 h, 50*C*=72 s). These achievements are promising and have resulted in commercially available batteries, for example, the automotive market; however, they are not sufficient to satisfy the ever-expanding needs for rechargeable lithium-ion batteries in all markets.

In general, lithiation and delithiation of LFP proceed by phase separation (two-phase coexistence)^1^. What was initially thought to be a fixed thermodynamic miscibility gap^7^,^8^ was later demonstrated to be particle size dependent by mean-field calculations by Burch^9^. This particle size dependence was experimentally validated by Wagemaker *et al*.^10^ for particle sizes less than a factor of ∼4.7 times the interfacial width, *λ* (ref. 9), or 57 nm (ref. 10) (based on an 80% miscibility gap). In addition to the two thermodynamically stable phases for LFP and FePO_4_ (FP), Delacourt *et al*.^11^ identified a metastable, temperature-driven solid solution in the LFP phase diagram; this solid solution forms intermediate phases of stoichiometry Li_0.75_FePO_4_ and Li_0.52_FePO_4_ on cooling at 1 K min^−1^, and these phases transform slowly into LFP and FP at room temperature. Chen *et al*.^12^ also reported that metastable phases with stoichiometries of Li_0.6_FePO_4_ and Li_0.34_FePO_4_ formed during cooling. One of these metastable phases (Li_0.6_FePO_4_) is equivalent to the concentration at the eutectoid point in the phase diagram at approximately 200 °C (ref. 8), whereas the other three proposed intermediate phases have no such presence in the phase diagram.

Recent research has demonstrated that the phase diagram for LFP depends not only on its thermodynamic properties but also on its kinetic properties. In 2008, Chang *et al*.^13^ performed the first high-rate *operando* synchrotron X-ray diffraction (XRD) for LFP at rates ranging from 1*C* to 10*C*. However, they observed no solid solution reaction for their large 3-μm particles^13^. This result was confirmed by Leriche *et al*.^14^ for LFP particles with sub-μm-to-μm sizes. Two years later, Kao *et al*.^15^ proposed that an overpotential-dependent amorphous phase might form in LFP. They showed that, for overpotentials <20 mV and >75 mV, a crystalline-to-crystalline phase separation should occur for a particle size of 113 μm, whereas at in-between overpotentials, a crystalline-to-amorphous phase transition might occur^15^. By contrast, Orikasa *et al*.^16^ recently reported a metastable phase with the composition Li_0.61–0.66_FePO_4_ during *operando* synchrotron XRD at a 10*C* rate for ∼400-nm particles (estimated from ref. 16). This intermediate phase diminished under open-circuit conditions, with an estimated lifetime of ∼30 min.

Very recently, two studies with similar experimental setups were published. Zhang *et al*.^17^ observed evidence for the onset of a solid solution phase during charging at 5*C* and 10*C*, whereas at 60*C*, a major portion of the electrode exhibited diffraction of intermediate phases. These results were confirmed by Liu *et al*.^18^ who showed that the intermediate Bragg reflections originate from the entire particle and not from the phase boundary alone. However, in both setups, the phase transitions occurred in the thickness direction of the composite electrode, which is also the direction of the XRD beam. Thus, a time-resolved deconvolution of the phase progression is not possible because this technique averages over the entire electrode thickness without focusing on the reaction front. Additionally, Zhang *et al*.^17^ proposed a free energy landscape that allows the formation of a preferred intermediate phase of Li_0.66_FePO_4_, as observed by Orikasa *et al*.^16^ However, they presented only minimal experimental evidence at the initiation of their 5*C* and 10*C* charging processes, as shown in ref. 17.

Several models exist to describe the phase separation behaviour of LFP. The shrinking-core idea was first outlined by Padhi *et al*.^1^ and was later refined by Srinivasan and Newman^19^. This idea was further developed by the observation of an anisotropic alignment of the phase boundary^20^ that formed a highly energetic interface between LFP and FP^21^. On the basis of these works, Singh *et al*.^9^,^22^ developed a one-dimensional model for LFP in 2008. Bai^23^ extended Singh's model and suggested an exchange current, density-dependent stability diagram for LFP, which showed phase separation at a low exchange current load, *i*/*i*_0_, a quasi-solid solution around the exchange current density, and true solid solution behaviour for LFP above a load of *i>*2*i*_0_ in Li_*x*_FePO_4_. By contrast, density-functional theory calculations performed by Malik *et al*.^24^ suggested the existence of a continuous solid solution pathway with no preferential phases.

The phase diagram and kinetic models of LFP are still actively discussed. This study aims to bring clarity to this discussion by investigating the phase diagram of LFP at high cycling rates. To address this issue, better *operando* methods are needed because the formation of intermediate phases is transient, non-equilibrium and unstable. First, an *operando* XRD–electrochemical impedance spectroscopy (XRD–EIS) technique is introduced and applied to LFP. Second, the results from the XRD-direct-current (XRD-d.c.) cycling are discussed. This work may support or disproves the various scenarios for the phase behaviour of LFP presented in the literature^15^,^16^,^17^,^18^,^23^,^24^. It may disprove the development of amorphous phases^15^ and the existence of a continuous current-independent, solid solution pathway^24^, but it lends support to the existence of intermediate metastable phases^17^,^18^ especially of composition Li_0.61–0.66_FePO_4_ (ref. 16) and the existence of an extended load-dependent solid solution^23^. These findings may explain why the charging times of LFP-based batteries are among the shortest known today.

## Results

### *Operando* XRD–EIS

**Figure 1:**
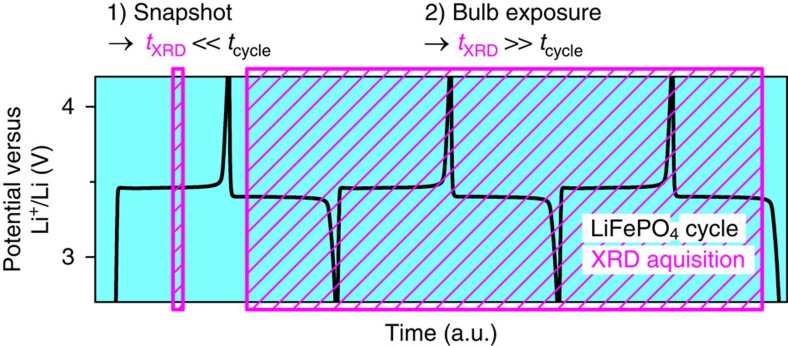
Principle of superposed XRD measurement. Bulb exposure technique applied here as a combined XRD–EIS technique using XRD exposure time *t*_XRD_ over several cycles (where *t*_cycle_ is the time for one cycle) to detect the traces of transient phases between the well-defined end phases of LFP and FP, in contrast to the standard XRD snapshot technique (cycling of LFP in black and exposure time in magenta).

Conventional operando diffraction methods cannot be applied at ultra-high charge/discharge rates because the shorter exposition time (*t*_XRD_) for snapshot XRD leads to a smaller signal-to-noise ratio (*S*/*N*) and, thus, to a weaker signal for phase identification. Therefore, superposition (that is, binning) of several XRD patterns can be performed to improve the *S*/*N* ratio^18^. Here, we propose a novel *operando* method that can be considered a ‘superposed XRD measurement', similar to photography with very long exposure times (for example, night photos of stars or traffic). The principle of XRD–EIS is relatively simple. As cycling times are decreased, bulb exposure can be applied to the XRD measurements, as shown in Fig. 1. The superposed XRD patterns show the traces of any phase transitions that occur during the respective charge/discharge processes. In the case of phase-separating materials such as LFP, the superposed information should be sufficient for phase identification because one inspects for intermediate phases between the well-known stable end phases of LFP and FP.

The necessary conditions for this measurement are completely opposite to those for standard *operando* XRD. The cycling time must be shorter than the acquisition period; thus, we can examine for any fast reactions at a *C*-rate>2/*t*_XRD_ to cover at least one full cycle. In this method, we can superpose as many XRD patterns as desired to increase the *S*/*N* ratio; however, the electrochemistry must be reproducible. In principle, information about the exact state-of-charge (SOC) of the electrode is exchanged for a greater detectable quantity of transient and metastable phases. A special pouch cell was designed for the XRD–EIS experiments^25^; this cell provides good electrical contact and high pressure on the electrodes to guarantee very fast repetitive cycling (Supplementary Fig. 1). An EIS analyser was used with high sinusoidal amplitude in the region of low frequency (this would correspond to the Warburg impedance regime at low excitation amplitudes). However, high-voltage EIS excitations of 500 mV would violate the condition of linearity around the open-circuit potential and could not be evaluated with standard Randles-type equivalent circuits. We used the EIS analyser simply as a source of ultra-fast, high power excitation.

**Figure 2:**
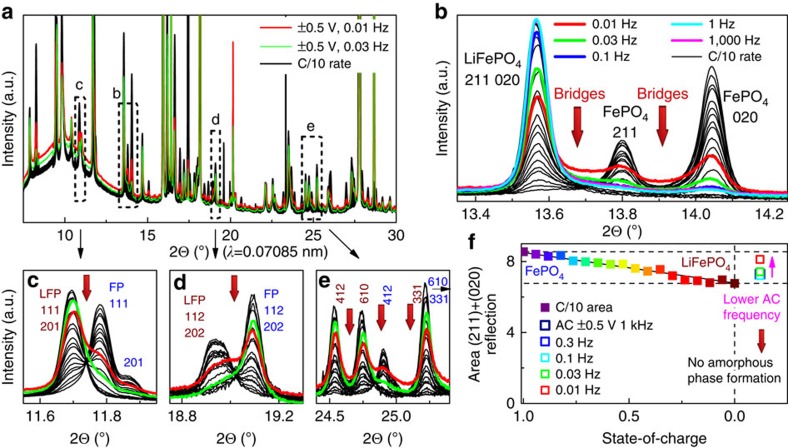
Evolution of *operando* XRD–EIS. Patterns at ±500 mV a.c. excitation for different frequencies (colours) in comparison to 0.1*C* low-rate XRD-d.c. patterns (black): (**a**) full-range XRD patterns; (**b**) solid solution bridges between LFP and FP for 0.01–1,000 Hz in comparison to the same electrode at a rate of 0.1*C*; (**c**–**e**) bridges appear over the entire measured 2*θ* range of 4–60° for 0.01- and 0.03-Hz XRD–EIS; (**f**) convoluted area of the (211) and (020) Bragg reflections of LFP at a rate of 0.1*C* in comparison with the XRD–EIS patterns at various frequencies for the same cell.

The application of XRD–EIS to LFP with high-to-low-frequency a.c. waves of 1,000 and 0.01 Hz is depicted in Fig. 2a,b. For example, a frequency of 1 Hz would correspond to a rate of 7,200*C*. Because this frequency was too high for diffusional processes within the LFP particles, the corresponding XRD patterns were almost identical to LFP at the initial state-of-charge (SOC=0). By contrast, for much lower frequencies of 0.03 and 0.01 Hz (≈216*C* and 72*C*, respectively), the active material started to react, as shown in Fig. 2b. The SOC changes during the first few low-frequency EIS excitations to an SOC, where the extracted specific charge during the positive and negative sine waves (lithiation and delithiation, respectively) sum to zero. Therefore, the Bragg reflections of LFP decreased with decreasing frequency, whereas those of FP increased because more charge could be extracted in each semi-sinusoidal wave (longer period).

‘Intensity bridges' appeared between the Bragg reflections of LFP and FP, indicating a continuous structural change. If we assume Vegard's law hold for the lattice parameters of the *Pnma* structure, as validated for LFP experimentally in the literature^11^,^26^,^27^ (Supplementary Note 1), then we propose that these ‘bridges' are clear signatures of the formation of a solid solution with a distribution of compositions. These ‘bridges' were not observed in the XRD patterns obtained at low *C*-rates; however, they were observed recently in XRD patterns at medium to high rates (5*C* to 60*C*)^17^,^18^. These in-between reflections were observed for the full XRD patterns over the 2*θ* range from 4 to 60°, as shown in Fig. 2c–e. The absence of maxima for the solid solution bridges during ultra-fast XRD–EIS cycling suggested the absence of preferred intermediate phases between LFP and FP at high rates. These high rates of up to 7,200*C* are very difficult to explore by the conventional XRD snapshot technique, even with binning of the XRD patterns.

No formation of amorphous phases was observed, which has been suggested to be accompanied by an area decrease of the Bragg reflections by Kao *et al*.^15^. Figure 2f shows the total area underneath all (211) and (020) Bragg reflections resulting from the XRD–EIS run in comparison with the total area at 0.1*C* rate. At any frequency, the accumulated areas were on the line towards FP because the SOC increased with decreasing frequency, as discussed above. Therefore, no evidence of amorphous phase formation was observed in these data, consistent with previous findings^17^,^18^.

### *Operando* XRD during galvanostatic cycling

**Figure 3:**
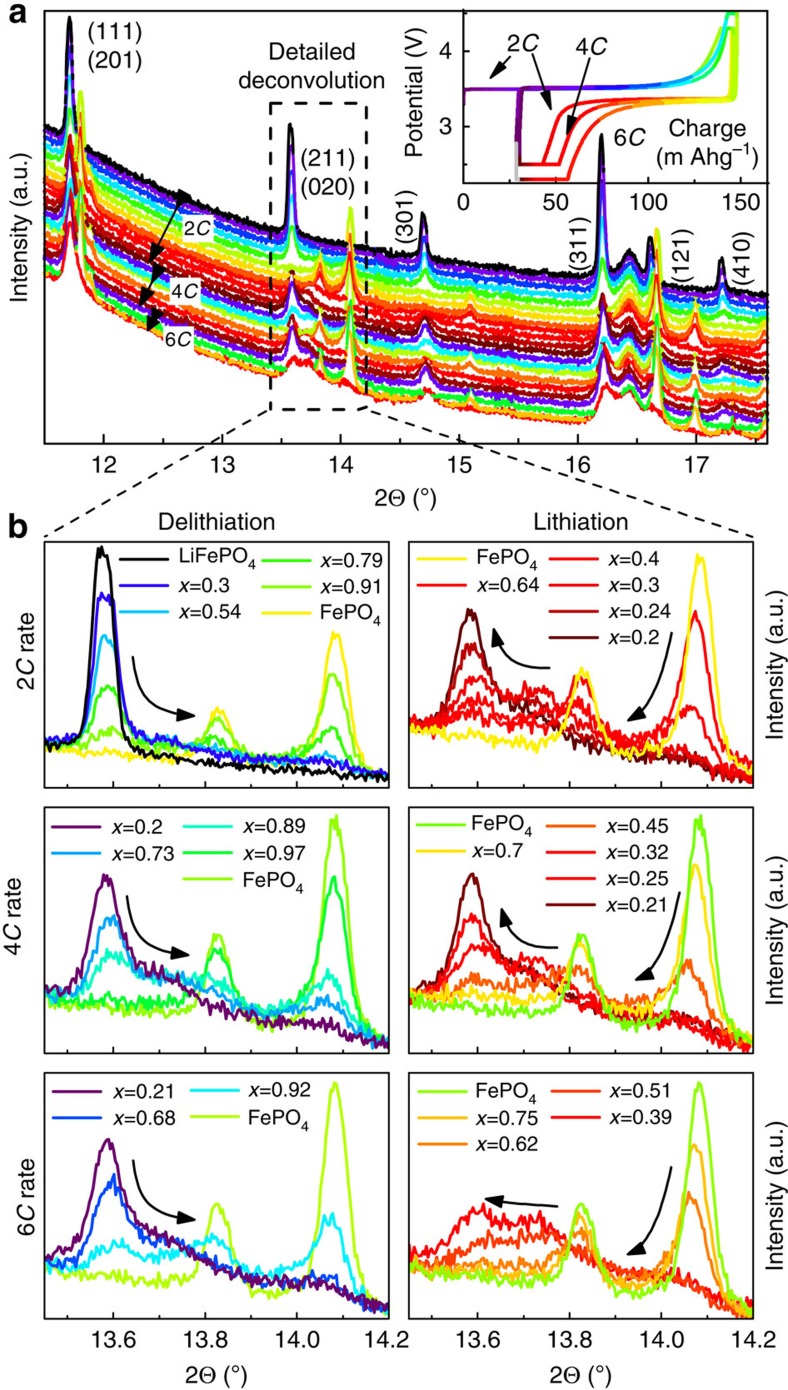
Evolution of *operando* XRD-d.c. (**a**) A thin-layer Li–LFP–Li three-electrode sandwich cell cycled at 2*C*, 4*C* and 6*C*; inset: electrochemical cycling; (**b**) zoomed and separated regions of the XRD patterns showing the phase evolution for lithiation and delithiation at individual rates (rainbow colours start for each cycle from violet for LFP to light green for FP and back to red for LFP for all rates).

In contrast to the *operando* XRD–EIS method, galvanostatic direct-current cycling can only be performed at low-to-medium *C*-rates^17^,^18^. In this study, rates of 2*C*, 4*C* and 6*C* were used in conjunction with a special cell design (Supplementary Fig. 2) to investigate the difference between the behaviours of LFP cycled at 0.1*C* and that of LFP cycled at ultra-high a.c. cycling rates (Supplementary Table 1). The diffraction patterns collected during d.c. cycling are depicted in Fig. 3a as a part of the full XRD pattern from 4° to 60°; the corresponding electrochemical cycling data are shown in the inset.

Magnification of the (211) and (020) Bragg reflections in Fig. 3b gives better insight into the phase transitions detected by this method. At a 2*C* delithiation rate, the LFP reflections decreased continuously as cycling proceeded. Small ‘intensity bridges' similar to those observed in the XRD–EIS results were also detected. However, during the first 2*C* delithiation (charge), the total intensity of the ‘bridges' was small compared with those of the LFP and FP reflections. For 2*C* lithiation (discharge), FP was observed to evolve into an intermediate phase with a concentration similar to that of FP; this intermediate phase then further transitioned into more lithium-rich intermediate phases and finally ended with the well-known LFP reflections at the end of discharge, as shown in Fig. 3b. Notably, these intermediate phases do not correspond to a single phase of a specific concentration, as concluded by Orikasa *et al*.^16^; rather, a phase transformation moves through the LFP electrode sideways with intermediate phases of all Li concentrations (*x*) in Li_*x*_FePO_4_, 0<*x*<1 during the transition.

The same trend is observed for 4*C* and 6*C* cycling in Fig. 3b in which the delithiation proceeded with the development of obvious intermediate phases. Therefore, the first medium-rate delithiation at 2*C* might activate the LFP particles to enable the formation of intermediate phases in subsequent cycles because the first 2*C* delithiation exhibits very little evidence of solid solution phases.

The intensity bridges detected by XRD-d.c. were also observed at 0.1*C*, as shown in Supplementary Fig. 3. The fraction of intermediate phases was always small at this low rate; however, the increased intensity of the background was obvious. We stress that 0.1*C* corresponded only to the globally applied current density (applied current divided by the total electrode mass). If only transient phases are is assumed to participate in Li exchange, the estimated local current density would be approximately five times higher because the total area of all intermediate phases underneath the (211) and (020) Bragg reflections was between 21 and 22% throughout the galvanostatic cycling. Therefore, for thick electrodes, a fraction of the LFP particles might bypass the usual two-phase reaction, even at 0.1*C*, and then undergo a solid solution reaction when inhomogeneous reactions increase the local current density.

### LeBail refinement of XRD-d.c. patterns

The qualitative evolution of the Bragg reflections is very prominent, as shown in Fig. 3. The refinements of the *operando* XRD patterns were difficult to interpret because inhomogeneous reactions and solid solution phases (broad reflections, many phases to refine simultaneously) were superposed on the electrode. We fixed this problem by constructing a cell to confine the inhomogeneous reaction into the lateral direction of the electrode so that the phase transition wave front moved into the beam spot sideways (see cell design in Supplementary Fig. 2).

Hence, the reaction front was delayed (Supplementary Fig. 4), but transient phases were allowed to occur along the total thickness direction of the electrode, which led to the detection of a large number of transient phases in the beam direction. In addition, the lateral phase movement allowed the determination of the phase evolution, which was not possible with the standard LFP electrode, in which the progression of the LFP/FP reaction front moved in the beam direction^16^,^17^,^18^.

The standard LeBail refinement^28^ was possible only for the end phases of LFP and FP because the lattice parameters of all intermediate phases were completely interchangeable. Hence, the XRD patterns were deconvoluted under the following conditions: (1) Vegard's law was applied which has been experimentally demonstrated to be valid in the case of LFP^11^,^26^,^27^. (2) The LeBail refinement of the FP pattern showed that the ratio between the (211) and the (020) reflection areas is 0.38. From the Inorganic Crystal Structure Database (ICSD) cards 72545 and 92199, the theoretical ratio was 0.17 and 0.29 for LFP and FP, respectively. Because the particles might be slightly aligned in the electrode, we assumed an area ratio between 0.23 and 0.38 (a factor of 1.3) for LFP and FP, respectively, with a linear combination for all intermediate phases. (3) The scattering factors of LFP and FP differ, as indicated by the total convoluted area in Fig. 2f. Therefore, all areas were normalized to the area of LFP by dividing the area of FP by a factor of 1.24 (derived from the end members in Fig. 2f). (4) The deconvolution of a pattern using an infinite number of phases was assumed to be impossible. Thus, we simply deconvoluted the patterns for the seven intermediate phases of composition Li_*x*_FePO_4_, *x*=(0.125:0.125:0.875) with the space group parameter being a linear combination of the refined end phases for LFP and FP (lattice parameters *a*, *b* and *c* for space group *Pnma* for LFP are 10.315(3), 6.000(2) and 4.687(2) Å, and those for FP are 9.811(9), 5.785(5) and 4.777(5) Å). All four assumptions were derived either from our experiments or from the literature and are further described in Supplementary Note 1. Because only nine different phases of SOC (0:0.125:1) were chosen to establish trends, this simplification assigned the other intermediate phases (for example, Li_0.57_FePO_4_) to the nine chosen ones. Using these assumptions, general trends can be extracted and productively used.

**Figure 4:**
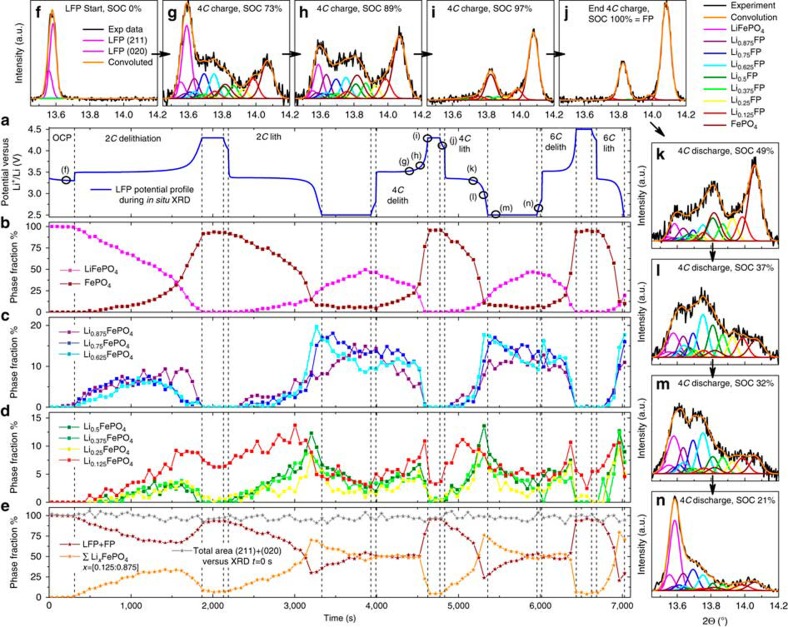
Deconvolution of *operando* XRD-d.c. Evolution of nine different phases of Li_*x*_FePO_4_ with *x*=(0:0.125:1), as calculated on the basis of Vegard's law for the LFP and FP end phases based on (211) and (020) Bragg reflections: (**a**) electrochemical cycling at 2*C*, 4*C* and 6*C*, with markers for deconvolution examples; (**b**) evolution of the LFP and FP phases, indicating the delayed reaction towards the end of each charge and discharge; (**c**,**d**) evolution of transient phases over the course of cycling, where the phases close to the starting phase evolve shortly after current is applied (note that transient phases remain at the end of discharge and transform very slowly during constant voltage and at open-circuit voltage, that is, the transient phase ‘freeze'); (**e**) sum of the end phases LFP+FP and all transient phases, showing the large amounts of intermediates of up to 70, 74 and 75% at the end of discharge at 2*C*, 4*C* and 6*C*, respectively; the total convoluted area during cycling (grey stars) was the same excluding the possibility of amorphous phases formation; (**f**–**n**) examples of deconvoluted *operando* XRD patterns during cycling marked in **a**).

The deconvoluted XRD patterns are shown in Fig. 4f–n. The phase transformation with the subsequent development of the intermediate phases in LFP can be observed. Three important observations are evident in Fig. 4a–e, where the fractions of the nine different phases are plotted versus the galvanostatic cycling profiles: (1) The phase transformation is delayed with respect to the average SOC because of confinement of the inhomogeneous reaction in the lateral direction; (2) the total fraction of all intermediate phases increased with increasing rate at the expense of LFP and FP. The quantity of intermediate phases reached its maximum shortly before the cutoff potential during discharge, which made up 70, 74 and 75% of the total XRD intensity for the materials discharged at rates of 2*C*, 4*C* and 6*C*, respectively, as depicted in Fig. 4e; and (3) the intermediate phase with a composition closest to the starting phase forms at the beginning of each charge and discharge and can represent as much as 14% of the total phase fraction. This fraction evolved only slightly during the first part of each charge and discharge. However, it transformed very rapidly through the other intermediate phases to the end phase at the final stage of each charge/discharge.

The XRD-d.c. experiments also showed that the total convoluted area of the (211) and (020) reflections (grey stars in Fig. 4e) changed only slightly during the course of cycling (s.d. 3.3%). These data eliminated the possibility of the formation of an amorphous phase. The overpotentials during charge and discharge at 2*C*, 4*C* and 6*C* were in the range for the proposed amorphous phase formation (here, 57 to 77 mV for charge, −75 to −129 mV for discharge; see Supplementary Table 2); therefore, we cannot support the finding by Kao *et al*.^15^ for the formation of an amorphous phase. However, the phase evolution of the end phases for LFP and FP in Fig. 4e seem very similar to those observed by Kao *et al*. (Illustration 6a in ref. 15). Illustration 7b in ref. 15 shows the same ‘intensity bridges' for the (211) and (020) Bragg reflections between 14.9° and 15.5°, whereas no background increase is observed between 15.6° and 16°. Thus, Kao *et al*. might have been the first to observe solid solution formation experimentally in 2010, initiating the discussion about high-rate dynamics in LFP^15^,^16^,^17^,^18^,^23^,^24^.

### Existence of preferred solid solution phases

To explain the differences observed between phase transitions detected at low, medium and high charge/discharge rates, two additional evaluations were conducted. The first evaluation focused on identifying preferred phases during the moment of phase transition of LFP to FP, whereas the second evaluation identified energetic barriers that hinder phase transformation.

**Figure 5:**
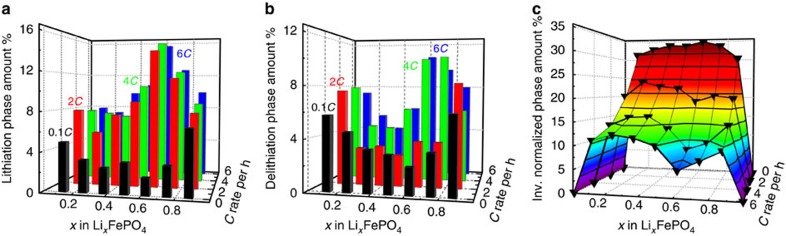
Preferred transient phases during transition and free energy. Amount of intermediate phases for (**a**) lithiation and (**b**) delithiation, averaged over 2, 3, 6 and 10 XRD patterns for 6*C*, 4*C*, 2*C* and 0.1*C* global rates. The XRD patterns were chosen so that sum of the phase fractions of LFP and FP match (Supplementary Table 2); (**c**) the inverse phase amount normalized with respect to LFP and FP end phases used to sketch the free energy as a function of the concentration and rate.

First, the quantity of intermediate phases that develop when the phase separation in the electrode moves through the beam spot can be directly evaluated. This is assumed to occur when the LFP and FP phases have the same total phase fraction in the XRD pattern. For 2*C*, 4*C* and 6*C*, we used 6, 3 and 2 XRD patterns corresponding to 40% of the SOC, where the sum of the fractions of LFP and FP were equal. These patterns were collected at the end of each galvanostatic charge and discharge step because of the delayed reaction. For 0.1*C*, we selected all patterns for discharge but only one for charge because of a beamline shut down. The XRD–EIS results could not be included because they do not fulfil the criteria of the same amount of LFP and FP phase (Supplementary Fig. 5). A plot of the total fraction of the intermediate phases during the phase progression, as depicted in Fig. 5, clearly revealed a preferred phase formation for Li_0.625_FePO_4_ for 2*C*, 4*C* and 6*C* rates. At 0.1*C*, no such preferred phase was observed; however, the intermediate phases close to the end members were the most stable. Only the 2*C* charge shown in Fig. 5b deviated, but this result might have been due to the activation of LFP, as discussed above.

If we take the average of the phase distributions for lithiation and delithiation and normalize them to the quantities of the end members for LFP and FP, we can determine the formation probability for each of these phases. This probability is connected to the free energy of the current-dependent stability diagram. Hence, we can interpret the inverse normalized phase amount in Fig. 5c as a sketch of free energy as a function of the composition and rate. The free energy function for 0.1*C* matched that proposed in the literature^29^. However, at higher rates, an intermediate phase with a composition close to the eutectoid point was preferred. Thus, the findings of an intermediate phase of Li_0.61–0.66_FePO_4_ reported by Orikasa *et al*.^16^ are supported, although we must stress that the phases around the eutectoid concentration were only slightly preferred to all other solid solution phases with compositions 0.125–0.5 and 0.75–0.875 and were not the only ones present, as previously proposed^16^.

**Figure 6:**
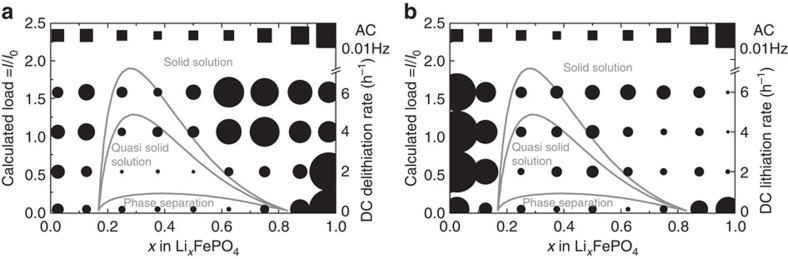
Avoided instability region during charge and discharge of LFP. Superposition of experimental results (black markers) with previous modelling results^23^ (grey lines, reprinted with permission from, Bai Cogswell Bazant, Nano Lett. 11, 4890. Copyright 2011 American Chemical Society, circles for XRD-d.c. and squares for XRD–EIS). The size of the markers represents the amount of phases integrated over the cycling period (galvanostatic part for XRD-d.c. or single XRD pattern at 0.01 Hz for XRD–EIS); intermediate phases (SOC=0.125–0.875) are plotted four times larger compared with pure LFP and FP to increase visibility; (**a**) for delithiation=charge; (**b**) for lithiation=discharge. The calculation in ref. 23 was performed only for discharge (no further information is available on charge, so the same stability diagram was used). The *y* axis of the *C*-rate and the calculated normalized load were matched arbitrarily.

The second evaluation was based on each phase fraction being integrated over the total galvanostatic region (for charge and discharge processes separately). This evaluation indicates the presence of preferred intermediate phases on particles that became ‘stuck' before they fully converted to the end phase, FP or LFP, at the end of charge or discharge, respectively. The sum of all fractions for the galvanostatic part and the XRD–EIS are summarized in Fig. 6. The results of the XRD-d.c. current experiments at 0.1*C*, 2*C*, 4*C* and 6*C* and the results of the XRD–EIS ultra-high-rate experiments partially support the theoretical model proposed by Bai *et al*.^23^. However, the exchange current density *i*_0_ (left *y* axis) and the global *C*-rate shown by our experiments (right *y* axis) are matched arbitrarily because inhomogeneous reactions in the electrode and the widely varying range of experimentally determined exchange current densities preclude us from drawing a firm conclusion.

As shown in Fig. 6a, the solid solution phases with high lithium content (here mimicked by Li_0.875_FP, Li_0.75_FP, and Li _0.625_FP) dominate as the delithiation rate increases. These phases formed shortly after the current was applied at each charge rate, as shown in Fig. 4c. The particles adhered to these phases. Only at the end of the galvanostatic charge these phases transform quickly through the preferred eutectoid concentration to form FP. During the potentiostatic step and open-circuit potential step, no intermediate phase was observed other than the remainder Li_0.125_FePO_4_, which might have been a small fitting artefact, as depicted in Fig. 4j.

However, during lithiation, only the Li_0.125_FePO_4_ phase formed shortly after the constant *C*-rate was applied, whereas the formation of all other phases was delayed until the end of the galvanostatic discharge. When the overpotential reached the cutoff potential (0.8–1 V total overpotential), the concentration of all the high-lithium phases had reached their maximum, whereas the concentrations of the low-lithium phases decreased to nearly zero (Fig. 4c,d). Even during the potentiostatic step, after a 10-min hold followed by a 1-min open-circuit potential step, the solid solution phases decreased very slowly despite the high overpotentials. During the subsequent charge, the total amount of high-lithium phases was approximately constant, whereas the amount of low-lithium phases increased. At the very end of the charge process, all solid solution phases quickly transformed to the end phase FP, as previously discussed. This results indicates a significant asymmetry between charge and discharge, where the intermediate phases seem to ‘freeze' at the end of discharge and where further filling from the intermediate phases with high-lithium concentration to the end phase LFP appears to become stuck, limiting the delivered specific discharge capacity.

When we integrated the phase amounts over the total time for each charge and discharge, we noticed that the formed phases tended to avoid the instability region calculated by Bai *et al*. (Fig. 6). However, the phases must pass through these instability regions at all rates. Thus, we can speculate whether the electrode would react homogeneously if the instability region in Fig. 6 did not hinder the continuous reaction inside the LFP particles. The phases form quickly after the current is applied, and they then stick at the energy barriers from the side where the concentration change is driven (from right to left for delithiation in Fig. 6a and from left to right for lithiation in Fig. 6b). At very high rates during XRD–EIS experiments, no such barrier was observed, as predicted by the calculations of Bai *et al*.^23^.

## Discussion

These findings substantially affect our understanding and ability to improve the performance of LiFePO_4_ electrodes used in some of today's lithium-ion batteries and desired for use in all markets. As solid solution phases are likely to form at medium-to-high *C*-rates, the interface area available for Li-ion insertion increases dramatically from 8 to 12 nm for the measured width of the phase boundary (Fig. 4 in ref. 30) to the total length of the particle in the a-direction, which is ∼150–200 nm, according to the results reported herein. This increase represents an almost 20-fold improvement. Thus, intercalation can proceed very rapidly, and high rates can be achieved with moderate overpotential because of the presence of a much larger active surface area of LFP particles exposed to the electrolyte.

In summary, a highly powerful tool was developed by combining high-resolution XRD and EIS to analyse metastable phases at high cycling rates in LFP cells. We confirmed previous findings and revealed several new insights using our new *operando* XRD–EIS and XRD-d.c. experiments. First, we observed the formation of solid solution phases in LiFePO_4_, confirming the results of previous work^16^,^17^,^18^,^23^; these phases enable the high-rate performance of LFP. Second, no amorphous phase formation was detected at any rate, contradicting previous findings^15^. Third, we observed equally distributed, solid solution phases at very high rates, a slightly preferred solid solution phase around the eutectoid concentration at medium rates of 2–6*C*, and small contributions of solid solution phases even at a very low rate of 0.1*C*, with preferred phases with compositions similar to those of LFP and FP.

From these experimental findings, we reconstructed a rate-dependent, free energy diagram indicating the preferred eutectoid phase. Furthermore, LFP avoids the rate-dependent instability region calculated previously^23^; however, the arbitrary match of exchange current and *C*-rate leaves uncertainty. In addition, the freezing of the intermediate phases at the end of discharge is prominent, which might shed some light on the asymmetry between charge and discharge of LFP in general, which greatly hindered the phase equilibration, even at a high overpotential of 1 V and during relaxation at the end of discharge. Further modelling of LFP is needed because the observed eutectoid phase and a shift of the free energy landscape with the rate are still open to theoretical exploration.

Additionally, our novel *operando* XRD–EIS method is expected to help identify transient phase formation during high-rate charge/discharge for other phase-separating battery materials such as LiMn_2_O_4_, Li_4_Ti_5_O_12_ or graphite because they also exhibit unusually high *C*-rate performance^31^,^32^.

## Methods

### Electrochemistry

Two slurries containing commercial LFP^33^ were prepared for use in the preparation of electrodes: (1) a slurry of LFP (Nippon Chemical, Japan), polyvinylidene fluoride binder (PVDF; Kynar Flex, Switzerland) and SuperP (Imerys, Switzerland) in a mass ratio of 70:15:15 in acetone; this slurry was cast onto a polytetrafluoroethylene foil to create self-standing electrodes for XRD-d.c.; and (2) the same materials in a ratio of 75:10:15 in 1-methyl-2-pyrrolidone for electrodes on Ti-foil current collectors^25^ for XRD–EIS. Electrodes were cutout and dried under vacuum at 120 °C for 12 h to remove any remaining water. The thickness for the XRD-d.c. electrodes was 43–45 μm to avoid diffusion limitations in the porous electrode. The electrodes had a mass of 4.35 mg and an electrode area of ∼3 cm^2^. The self-standing LFP electrode was squeezed between two Al-meshes with an 8-mm diameter hole in the centre to avoid Al Bragg diffraction. The LFP electrode for the XRD–EIS consisted of 4.64 mg of LFP with a thickness of 32 μm, giving a porosity of ∼49% of the volume. In both cells, 2 ml of 1 M LiPF_6_ in ethylene carbonate: dimethyl carbonate (1:1 wt; Novolyte) was used as an electrolyte. Scanning electron microscopy images of the LFP electrodes are shown in Supplementary Fig. 6. The particle size of a single carbon-coated LFP particle was ∼150–200 nm. These single particles were often agglomerated into small networks or clusters.

### Pouch cells

Two different pouch-cell configurations, one for XRD-d.c. and one for XRD–EIS, were employed. Pouch cells for the XRD-d.c. experiments were designed to minimize ionic diffusion limitations and to increase the XRD *S*/*N* ratio by using non-crystalline materials in the diffracting beam. The cells were prepared from polyethylene-coated aluminium bags equipped with an 8-mm Kapton (polyimide) and polypropylene window in the centre. The combination of polyimide and polypropylene is important because the average oxygen permeation rates are 35 and 900 cm^3^ O_2_ per m^2^ per 24 h 0.1 mm^−1^, respectively, whereas the corresponding moisture permeation rates are 20 g and 3 g H_2_O per m^2^ per 24 h 0.1 mm^−1^ for Kapton and non-oriented polypropylene, respectively^34^. Two Li counter electrodes were used in a Li–LFP–Li sandwich configuration. This configuration was used to decrease the diffusion length within the porous LFP electrode by a factor of two, thus offering the following advantages: pure crystalline reflections of LFP, very high reaction kinetics, and a homogenous reaction in the thickness direction (beam direction) of the electrode. However, amorphous scattering contributed to the background at low 2*θ* angles because of the presence of polymers. Pouch cells for the XRD–EIS experiments were constructed to apply high pressure to the electrodes. Therefore, two 50-μm thick titanium foils were used as XRD windows and current collectors simultaneously, allowing the cell to operate for more than a week. However, these foils decreased the intensity by 63%. The addition of Ti Bragg reflections made the full pattern not overlap with that of LFP in the 2*θ* range of interest. Both pouch cell types were assembled using a vacuum-sealing device in an argon-filled glove box from which N_2_, O_2_, H_2_O and organic volatiles were continuously removed. A detailed description of the cell configuration is available in Supplementary Figs 1 and 2 and in ref. 25.

### *Operando* synchrotron XRD and cycling

*Operando* XRD patterns were collected at the material science beamline-X04SA at the Swiss Light Source in Villigen, Switzerland^35^. The XRD patterns were collected in transmission mode at a wavelength (*λ*) of 0.7085 Å using a Mythen II detector. The resolution of the detector was 0.00376°; however, a 200-μm capillary provided an actual resolution of 0.0188° when the detector was placed 761.5 mm away from the sample^35^. The actual beam was 0.5 × 0.5 mm^2^. A VMP3 (Biologic) was used for d.c. and EIS excitation. The XRD exposure time was set to 40 s and 2 min for the medium-rate d.c. and high-rate a.c. experiments, respectively. The end phases LFP and FP were refined using the standard LeBail refinement (Supplementary Fig. 7). The intermediate phases Li_*x*_FePO_4_ (*x*=0.125 to 0.875) were refined starting from 0.125 to 0.875 subsequently where the unit-cell parameters of these phases are a linear combination of the end phases based on Vegard's law. The peak width was also refined but was limited by lower and upper bounds. When all peaks where refined, iteration was conducted. Usually two to three iterations were performed. Convergence was assumed to be reached when the change of peak area was <1%. For the EIS experiments, potential sine-wave amplitudes of 0.2 or 0.5 V were applied in a frequency range of 1 kHz–0.01 Hz so that the XRD exposure time was always longer than the EIS period. The central potential of the sine wave was 3.435 V. The corresponding starting SOC was nearly 0%.

## Additional information

**How to cite this article:** Hess, M. *et al*. Combined *operando* X-ray diffraction–electrochemical impedance spectroscopy detecting solid solution reactions of LiFePO_4_ in batteries. *Nat. Commun.* 6:8169 doi: 10.1038/ncomms9169 (2015).

## Supplementary Material

Supplementary Information Supplementary Figures 1-7, Supplementary Table 1-2, Supplementary Note 1 and Supplementary References.
