# Supplementary material for: Combined operando X-ray diffraction–electrochemical impedance spectroscopy detecting solid solution reactions of LiFePO4 in batteries
Source: Nat Commun. 2015 Sep 8;6:8169. doi: 10.1038/ncomms9169 (PMC4569719; doi:10.1038/ncomms9169)
Supplement: Supplementary Information — Supplementary Figures 1-7, Supplementary Table 1-2, Supplementary Note 1 and Supplementary References. [file ncomms9169-s1.pdf]

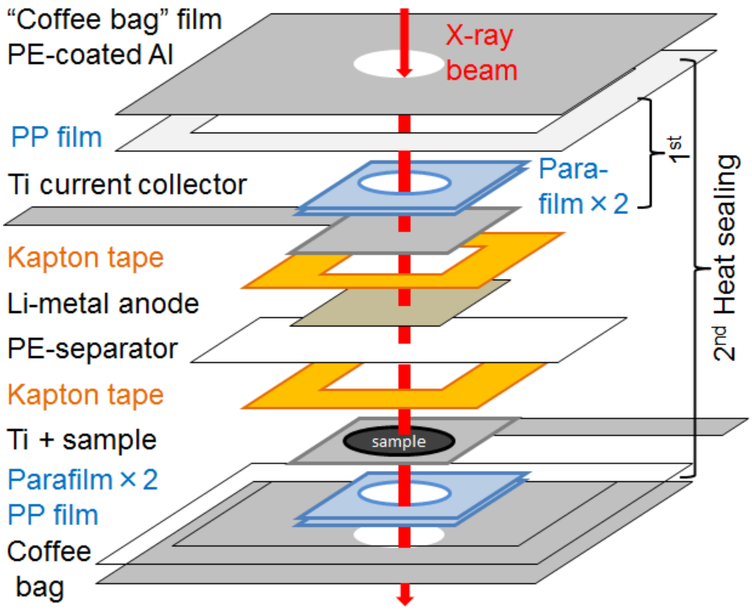

Supplementary Figure 1: Sketch of XRD-EIS pouch cell design with Titanium current collectors serving as XRD windows, para-film, kapton tape made from polyimide used to seal Titanium (Ti) current collectors and Aluminum (Al) pouch cell while poly-propylene (PP) and polyethylene (PE) used for sealing outer parts of pouch cell.

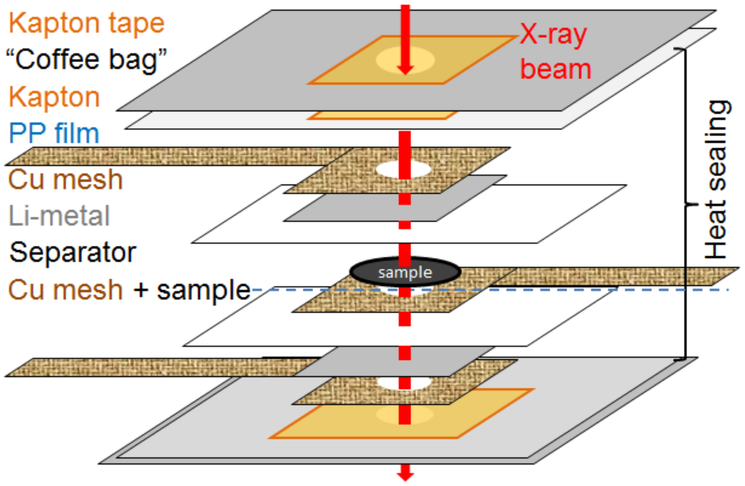

Supplementary Figure 2: Sketch of XRD-DC pouch cell with Cu mesh as current collectors with hole in centre avoiding Bragg reflections, sample sandwiched between two Li-counter electrodes, XRD window based on Polyimide (Kapton) and polypropylene (PP) blocking air gases and moisture, respectively. Cell is symmetrical around the sample in the centre.

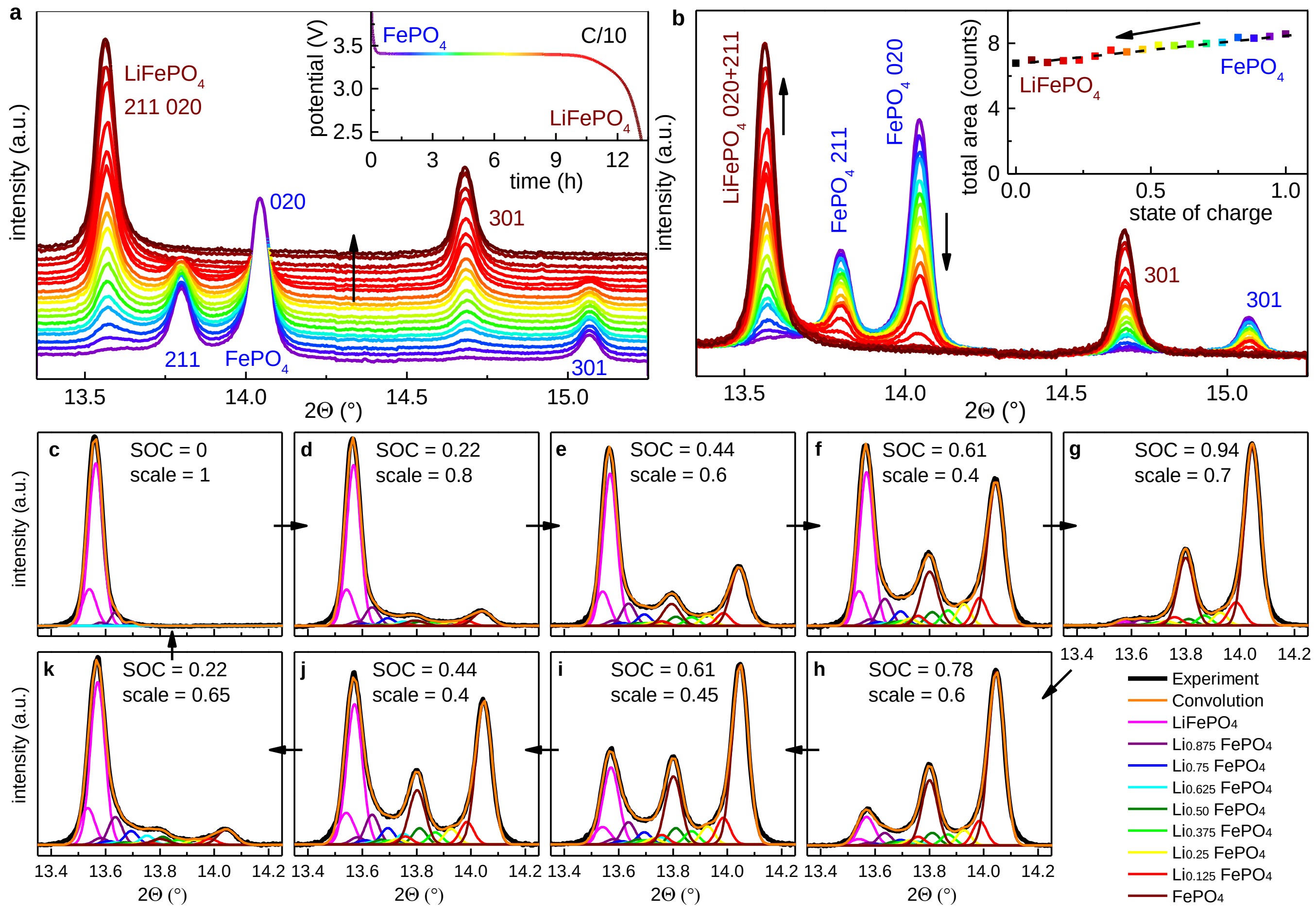

Supplementary Figure 3: ***Operando*** **XRD patterns collected during the C/10 discharge of $LiFePO_4$**, (**a**)waterfall XRD patterns with electrochemical discharge curve of LFP from 4.1 and 2.4 V in inset, (**b**) XRD patterns with total area in range 2Θ=13.4-14.2° in inset, color of each XRD pattern corresponds to the color of the discharge curve, (**c**)-(**k**) evolution of LFP (211) and (020) Bragg reflections cycled at C/10 charge and discharge, (**c**)-(**f**) delithiation with obvious intensity bridges, (**g**)-(**k**) lithiation; diffraction pattern of (**f**) and (**j**) are very similar despite the very different SOC indicating charge storage in intermediate phases leading to the observation of a pseudo delayed reaction even when the phase separation front moves in beam direction in XRD-EIS cell at low C/10 rate.

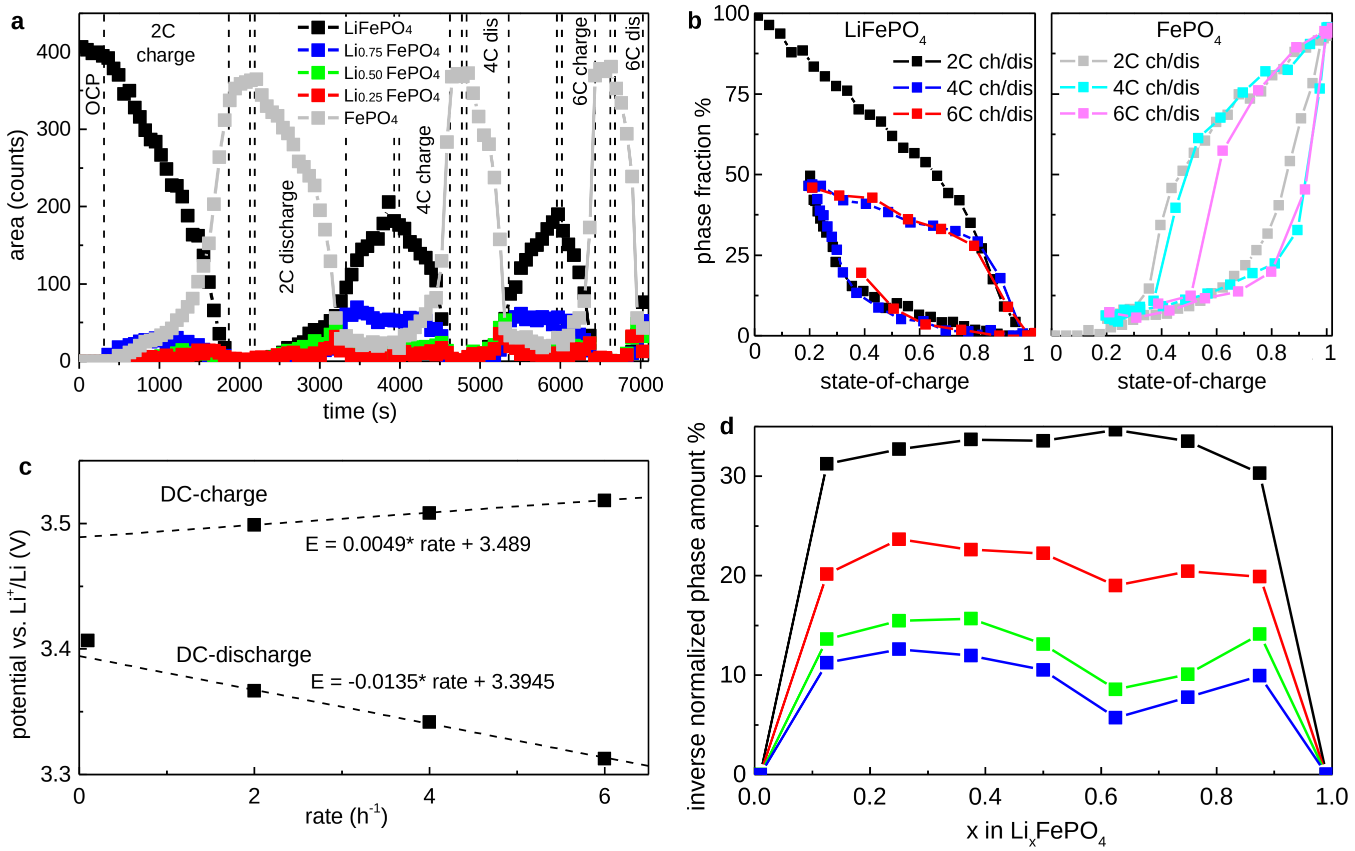

Supplementary Figure 4: Operando XRD-DC (**a**) Evolution of phases over the course of cycling for 2-6C XRD-DC, (**b**) *operando* XRD zoom of 2, 4, and 6C XRD-DC pattern as a zoom of Fig. 4 in main manuscript (MM), evolution of end phases (LFP and FP) during the medium rate DC cycling show delay of the local SOC determined from XRD to the electrochemical SOC (x-axis) as intended by the cell design for XRD-DC cell in Supplementary Figure 2, (**c**) overpotentials at 25% progression of each phase transition for 0.1, 2, 4, and 6C direct-current, fitted lines exclude 0.1C rate since experiment performed in XRD-EIS cell (Ti window, lower ohmic resistance), (**d**) Inverse phase amount of transient phases normalized versus LFP and FP end phases for different C-rates (corresponds to 3D plot of Fig. 5c in MM).

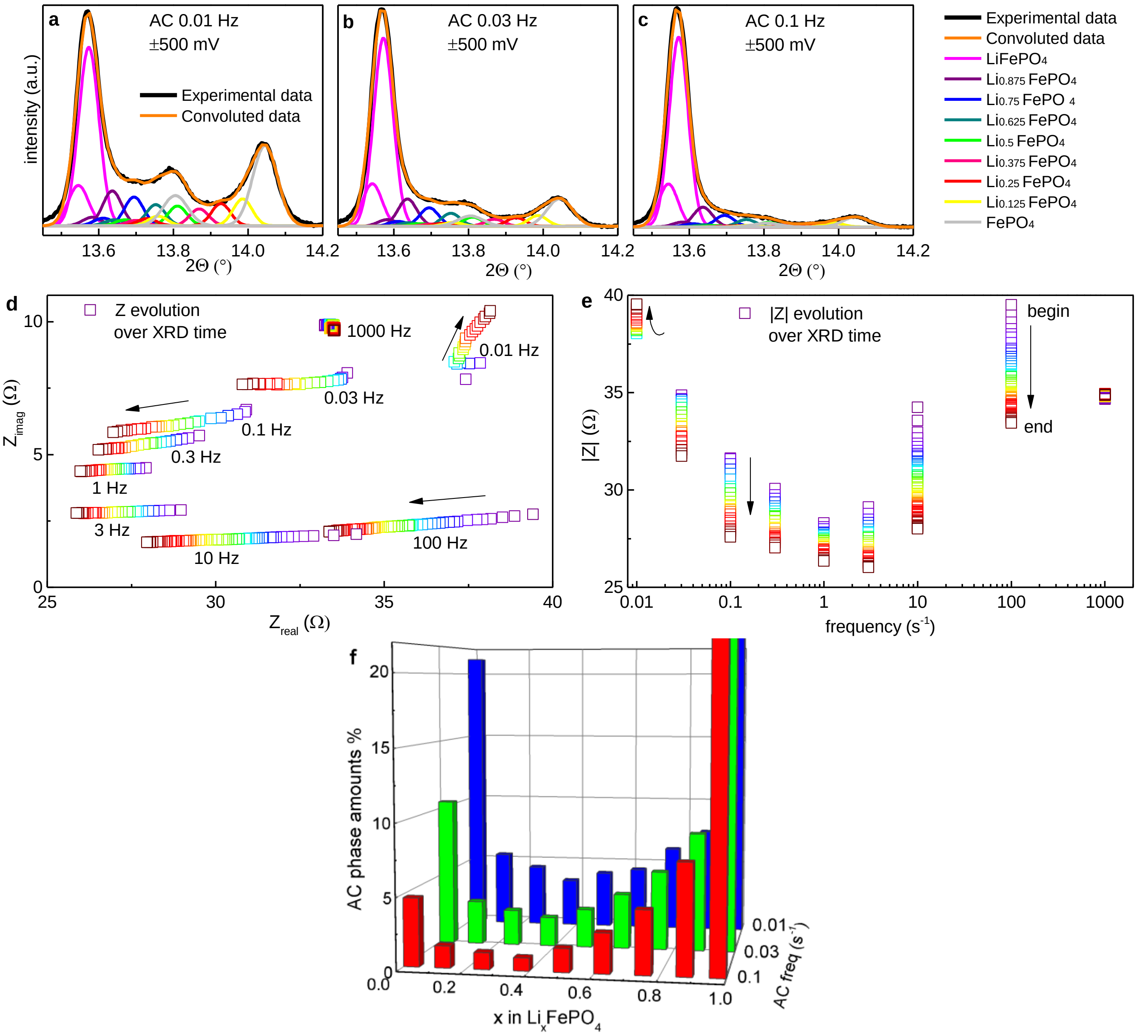

Supplementary Figure 5: ***Operando*** **XRD-EIS data.** (**a**)-(**c**) Deconvolution of XRD-EIS pattern for 0.1 to 0.01 Hz with nine different phases of composition LFP, FP and every SOC variation of 12.5 %, (**d**) Nyquist plot of XRD-EIS at various frequencies, (**e**) evolution of absolute impedance over XRD measurement time, (color corresponds to evolution of impedance Z over EIS excitation time starting from purple (begin) to dark red (end)), (**f**) Phase distribution diagram for XRD-EIS results indicating small bias between different phases which might be due to mixing with low rate currents as sinusoidal voltages excitation also crosses zero volt overpotential.

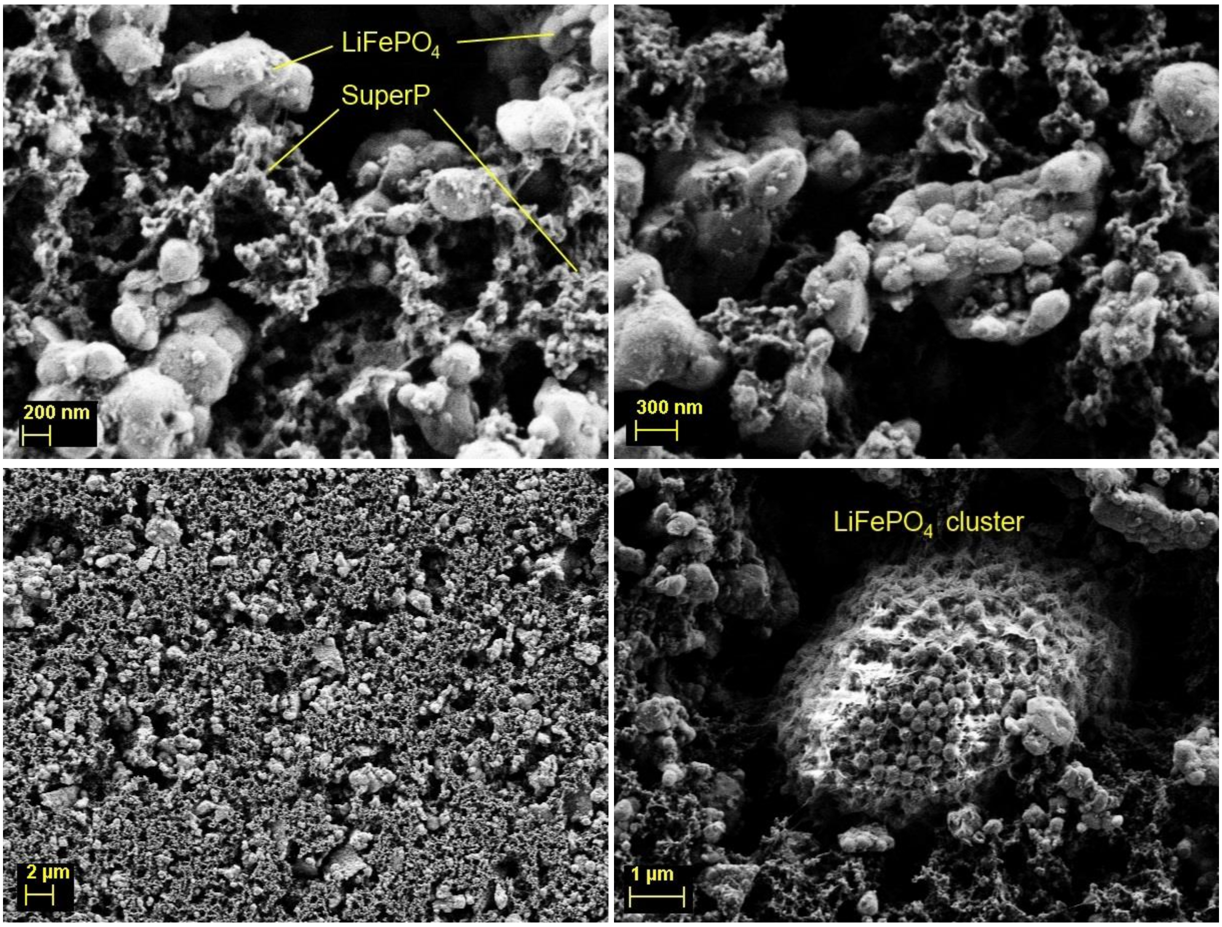

Supplementary Figure 6: SEM images of carbon-coated commercial $LiFePO_4$ electrodes with ratio LFP: PVDF: SuperP ratio of 70:15:15 for XRD-DC experiments, LFP primary particle often agglomerated to little networks (upper right) or even clustered (lower right), particle size around 150-200 nm primary particle size, well dispersed 15 wt% SuperP carbon black conductive additive, homogeneous porosity to ensure high ionic diffusion in the electrolyte.

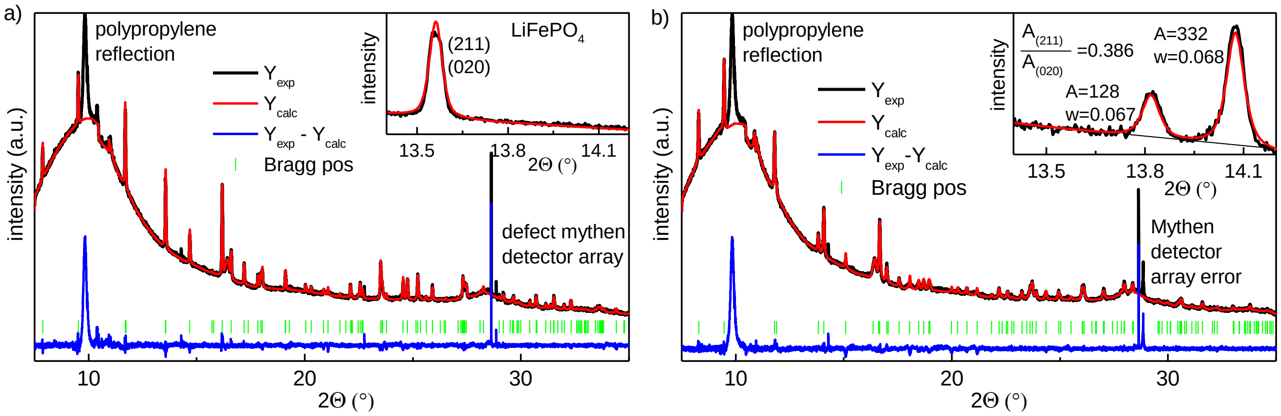

Supplementary Figure 7: Lebail refinement of (**a**) $LiFePO_4$ and (**b**) $FePO_4$ in Li-LFP-Li sandwich cell for XRD-DC measurements before and after 2C measurement at RT.

Supplementary Table 1: Comparison of cell design for XRD-EIS and XRD-DC pouch cell where XRD-EIS cell with Titanium current collectors is designed to supply good pressure for the impedance spectroscopy measurements at ±500 mV excitation and XRD-DC is design as sandwich Li-LFP-Li cell to have fast ionic diffusion and hole in Cu-mesh current collector to have limiting electronic conductivity to confine phase transformation in lateral direction (sideways into the XRD beam), XRD window is Ti foil for XRD-EIS cell and polyimide (PI), polypropylene (PP) and Li foil for XRD-DC cell.

	**XRD-EIS cell**	**XRD-DC cell**
Sketch	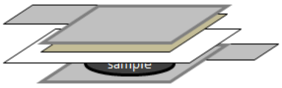	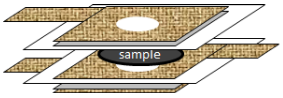
Counter electrode	One Li-counter electrode	Two Li-counter electrodes
Electronic cond.	Good electronic conductivity with plain Ti current collector	Limiting e- cond. In hole of Cu mesh current collector
Ionic cond.	Limiting ionic cond.	Good ionic cond.: Li sandwich structure, 44µm thick [illegible]
Progression of phase transformation in electrode	in thickness direction of electrode	in lateral direction of electrode
XRD window	Ti metal	PI+PP+Li metal

Supplementary Table 2: Deconvoluted phase fractions. (**a**) Contribution of phases during galvanostatic part of XRD-DC at 2, 4, and 6C charge/discharge and XRD-EIS at 0.01, 0.03 and 0.1 Hz and C/10 XRD-DC experiments, (**b**) phase contribution at moment of phase transition corresponding to Fig. 5c in MM and Supplementary Figure 6.

**a**	Delithiation (charge)				Lithiation (discharge)				XRD-EIS at various frequencies		
DC/AC rate	C/10	2C	4C	6C	C/10	2C	4C	6C	0.01Hz	0.03Hz	0.1Hz
$LiFePO_4$	59.7	57.8	30.9	32	37.4	6.9	4.4	5.5	42.6	60.9	75.1
$Li_{0.875}FePO_4$	7.3	5.5	9.1	9.3	6.6	3.6	3.5	4.4	7.7	8.5	7.7
$Li_{0.75}FePO_4$	3.4	4.3	11.1	10.6	3	2.6	2.1	5.3	6.2	5.7	4.4
$Li_{0.625}FePO_4$	1.7	3.4	9.4	11.5	1.8	3.7	3.9	5.9	4.6	3.9	2.8
$Li_{0.50}FePO_4$	2.3	1.3	4.3	5.7	3.1	4.4	5.2	5.6	4.2	2.8	1.6
$Li_{0.375}FePO_4$	2.2	1.4	4.2	3.3	2.6	4	4.1	4.8	3.5	2.1	0.9
$Li_{0.25}FePO_4$	2.7	1.3	2.7	4.1	3.3	2.6	3.8	4.3	4.6	2.5	1.2
$Li_{0.125}FePO_4$	3.3	4.2	7.1	6.2	5	9.7	9.3	7.6	5.5	3.1	1.5
$FePO_4$	17.4	20.8	21.2	17.3	37.2	62.5	63.7	56.6	21.1	10.5	4.8
overpotential	-	+57	+66	+77	-35	-75	-100	-129	±500	±500	±500

fractions in %, overpotentials in mV

**b**	Delithiation (charge)			Lithiation (discharge)		
DC rate	2C	4C	6C	2C	4C	6C
$LiFePO_4$	34.2	26.7	24.4	21.7	16.2	13.2
$Li_{0.875}FePO_4$	8.3	6	8	7.8	8.6	9.9
$Li_{0.75}FePO_4$	3.6	10.5	9.6	11.4	12.2	12.6
$Li_{0.625}FePO_4$	3.6	10.3	10.7	14.2	15.4	15.5
$Li_{0.50}FePO_4$	2.5	6	6	8.9	10.6	10.7
$Li_{0.375}FePO_4$	3.1	4.4	4.1	7.5	7.1	9.7
$Li_{0.25}FePO_4$	3	4.5	5.2	5.6	7.4	7.3
$Li_{0.125}FePO_4$	7.6	7.9	7.4	7.9	7.7	7.8
$FePO_4$	34.2	26.6	24.6	21.7	16.2	13.2

6, 3, and 2 XRD patterns are summed for 2, 4 and 6C rate so that total amount of LFP equals FP phase fraction

Supplementary Note 1: Validation of assumptions for deconvolution

Vegard's law

Besides the finding of several different intermediate phases in $LiFePO_4$ as discussed in the main manuscript (MM), the evolution of the lattice parameters is crucial. For the solid-solution reaction of Li_xFePO_4 at 350°C, Delacourt et al.[1] found a linear contraction of the a and b lattice parameters of -3.7 and -5.5% while the c parameter expanded non-linear by 1.4% in the Pnmb space group. In contrast, the refinement of the intermediate phases $Li_{0.6}FePO_4$ and $Li_{0.34}FePO_4$ by Chen et al.[2] showed strong deviation from Vegard's law. However, the 13 nm particles obtained by low temperature synthesis of $LiFePO_4$ of Gibot et al.[3] showed a linear relationship of all three lattice parameters a b c with slightly lower total expansion and contraction of -4.2, -2.5, and +1.4% (based on Pnma) which are slightly lower than those of Delacourt due to only 80% extracted specific charge. Also, the results of Sharma et al.[4] for deep-discharge LFP, indicate a linear relationship of the lattice parameters at room temperature (RT). Thus, only the results of Chen et al.[2] and the c lattice parameter in ref.[1] show a deviation from Vegard's law but are performed during cooling experiments. All measurements performed solely at RT follow strictly Vegard's law. Therefore, Vegard's law will be used for the deconvolution of the XRD patterns as it seems to be valid for solid-solution phases in $LiFePO_4$ at RT.

Area ratio of (211)/(020) Bragg reflections of $LiFePO_4$

The second assumption was based on the area ratio of the (211) to (020) Bragg reflections to allow a proper deconvolution. Therefore, the end phases LFP and FP have been refined for the lattice parameters in Suppl. Fig. 7. The zoom in the inset shows the area ratio of the (211) to (020) Bragg reflection. The refined space group parameters are 10.315(3) 6.000(2) 4.687(2) for LFP and 9.811(9) 5.785(5) 4.777(5) for FP, respectively.

Supplementary references:

1. Delacourt C, Poizot P, Tarascon J-M, Masquelier C. The existence of a temperature-driven solid solution in LixFePO4 for 0 < x < 1. *Nat Mater* **4**, 254-260 (2005).
2. Chen G, Song X, Richardson TJ. Metastable solid-solution phases in the LiFePO4/FePO4 system. *J Electrochem Soc* **154**, A627-A632 (2007).
3. Gibot P*, et al.* Room-temperature single-phase Li insertion/extraction in nanoscale Li(x)FePO4. *Nat Mater* **7**, 741-747 (2008).
4. Sharma N*, et al.* Direct Evidence of Concurrent Solid-Solution and Two-Phase Reactions and the Nonequilibrium Structural Evolution of LiFePO4. *Journal of the American Chemical Society* **134**, 7867-7873 (2012).
